# Male Sex and Ageing are Independent Risk Factors for Sarcopenia Stage in Patients With Chronic Kidney Disease Not Yet on Dialysis

**DOI:** 10.1002/jcsm.13612

**Published:** 2024-10-01

**Authors:** Yi‐Fang Huang, Shih‐Ping Liu, Chih‐Hsin Muo, Chen‐Yi Lai, Chung‐Ta Chang

**Affiliations:** ^1^ Department of General Dentistry Chang Gung Memorial Hospital Linkou Taiwan; ^2^ Graduate Institute of Dental and Craniofacial Science, College of Medicine Chang Gung University Taoyuan Taiwan; ^3^ School of Dentistry, College of Oral Medicine Taipei Medical University Taipei Taiwan; ^4^ Translational Medicine Research Center China Medical University Hospital Taichung Taiwan; ^5^ Program for Aging, College of Medicine China Medical University Taichung Taiwan; ^6^ Management Office for Health Data China Medical University Hospital Taichung Taiwan; ^7^ Internal Audit Department Far Eastern Memorial Hospital New Taipei Taiwan; ^8^ Graduate Institute of Medicine Yuan Ze University Taoyuan Taiwan; ^9^ Department of Emergency Medicine Far Eastern Memorial Hospital New Taipei Taiwan

**Keywords:** ageing, AWGS 2019, chronic kidney disease, gender, older adult, sarcopenia

## Abstract

**Background:**

The risk of sarcopenia in older adults with chronic kidney disease (CKD) not yet on dialysis is controversial. The aims of this study were to investigate the association among sarcopenia, diabetes and predialysis CKD and evaluate the impact of gender and ageing on the risk of sarcopenia statuses in older patients with predialysis CKD.

**Methods:**

The participants aged **≥**60 years old were recruited from the community of New Taipei City, Taiwan. Handgrip strength, appendicular skeletal muscle mass and the 6‐m walk were measured. The diagnosis of sarcopenia was established based on the consensus of Asian Sarcopenia Working Group 2019. These older adults were categorised into G1, G2 and G3–5 according to the guidelines of Kidney Disease Improving Global Outcomes (KDIGO) after calculating the estimated glomerular filtration rate by the Modification of Diet in Renal Disease equation. The Chi‐square test and ANOVA were used to estimate the difference of categorical and continuous variables, respectively. Polytomous logistic regression was employed to assess the odds ratio (OR) and 95% confidence intervals (CIs) of the sarcopenia status and sarcopenia‐associated risk factors in the predialysis CKD patients. All tests were two‐sided, and *p* < 0.05 was defined as statistical significance.

**Results:**

Among the 3648 older adults (mean age: 71.9 ± 6.07 years), including 1701 males and 1947 females, 870 (23.9%), 94 (2.58%) and 48 (1.32%) had possible sarcopenia, sarcopenia and severe sarcopenia, respectively. After adjustment, the risk for possible sarcopenia, sarcopenia and severe sarcopenia significantly increased with ageing (OR = 1.11, 1.10 and 1.23; 95% CI = 1.10–1.13, 1.07–1.15 and 1.18–1.30, respectively) and male gender (OR = 2.26, 20.3 and 25.4; 95% CI = 1.87–2.73, 11.5–36.0 and 11.3–57.2, respectively). Compared with KDIGO G1, no significant association between KDIGO G3–5 and the statuses of sarcopenia was observed (OR = 0.97, 0.88 and 0.91; 95% CI = 0.75–1.26, 0.43–1.78 and 0.37–2.27, *p* = 0.821, 0.718, 0.838, for possible sarcopenia, sarcopenia and severe sarcopenia, respectively). Ageing and male gender indicated a significant risk for higher sarcopenia status in older patients with predialysis CKD (0.027‐fold/year and 0.284‐fold, respectively) (*p* < 0.0001).

**Conclusions:**

This study illuminated the importance of the male sex and the ageing process on the risk of sarcopenia progression in patients with predialysis CKD. Early clinical screening and aggressive treatment for the prevention of higher sarcopenia status in advanced older male adults with predialysis CKD are recommended.

## 1 Introduction

According to the consensus of the Asian Working Group for Sarcopenia (AWGS) 2019 [1], sarcopenia is a skeletal muscle disease characterised by low muscle strength with a loss of muscle mass and/or low physical performance. Patients with sarcopenia have a higher risk of disability, fractures, hospitalisation, poor quality of life and mortality [2, 3]. Sarcopenia also occurs with several systemic diseases, such as metastatic cancer, diabetes mellitus (DM), heart failure and end‐stage kidney disease (ESKD) [4, 5, 6]. In all these conditions, the concurrence of sarcopenia predicts a remarkably higher risk for adverse prognosis. Recognising the associated risk factors of sarcopenia and initiating early surveys for disease prevention and treatment are imperative for health promotion in older adults.

In the human body, skeletal muscle is the primary storage of protein and provides amino acids for various organs to produce energy, with decreasing muscle protein synthesis and increasing inflammation and insulin resistance, subsequent muscle fibre shrinkage could occur. These catabolic processes can occur due to chronic illnesses, such as chronic kidney disease (CKD) and DM [7], but the clinical relationship between sarcopenia and older adults with CKD not on dialysis is still a controversial subject. Foley et al. reported a stepwise increase in the prevalence of sarcopenia with declining glomerular filtration rate (GFR) levels [8]. D'Alessandro et al. investigated that advanced older CKD males had a higher sarcopenia prevalence than younger participants [9]. However, the following two recent studies displayed different conclusions from the former reports. In 2020, a study based on the revised version of the European Consensus on Definition and Diagnosis of Sarcopenia (EWGSOP2) showed that the prevalence of sarcopenia does not increase with CKD severity [10]. In 2021, after a 16‐year longitudinal prospective cohort study by Lee and colleagues, CKD displayed no correlation with muscle loss [11]. Therefore, a large sample size research to determine the impact of predialysis CKD on the risk for sarcopenia with the latest diagnostic criteria of sarcopenia is warranted.

Since several new findings of sarcopenia challenge our current knowledge, the diagnostic criteria and definition of this disease are progressing. The clinical value of muscle strength evaluation is emphasised. Speed of muscle strength decrease presents two to five times greater than muscle mass loss [12]. Low muscle strength, regardless of muscle mass, is concluded as an independent risk factor for higher all‐cause, cardiovascular and cancer mortality in older adults [13]. For the purpose of recognising the patients with the risk of sarcopenia earlier, a new entity of ‘possible sarcopenia’ was defined in the diagnostic criteria of the AWGS 2019, which focused on the low handgrip strength older adults, to initiate exercise and diet modifications for this population [1]. There are also obvious differences in some cutoff values between the diagnostic criteria of AWGS 2014 and AWGS 2019 that may modify the current sarcopenia prevention and treatment strategies. Consequently, a reassessment of the association between sarcopenia and predialysis CKD is required.

CKD is one of the most common complications and concomitant conditions of DM [14]. Both these diseases have a high prevalence in older adults, but the synergistic effect of DM and CKD on the risk of sarcopenia in older subjects is not well established. There is also not enough evidence related to the impact of gender and ageing on the risk of sarcopenia in older adults with CKD. In this study, we aimed to examine the relation between predialysis CKD and different statuses of sarcopenia and the synergism of CKD and DM on the risk of sarcopenia according to the consensus of AWGS 2019. The impact of gender and ageing on the development of sarcopenia in older adults with predialysis CKD was also investigated.

## 2 Methods

### 2.1 Participants

We conducted a cross‐sectional study with community‐dwelling older individuals aged 60 years and above from New Taipei City, Taiwan, from 2015/01/01 to 2022/12/31. The exclusion criteria included dementia, Parkinson's disease, stroke, severe cognitive impairment, cachexia, disability and missing data. Since sarcopenia is not associated with body weight loss, any participant with a body weight loss of more than 5% in the past 12 months or less, or a body mass index < 20.0 kg/m^2^, was considered as a candidate for cachexia and was excluded [15]. A total of 3648 eligible older adults were enrolled in the study after they had signed an information consent form. This study was executed in accordance with the Declaration of Helsinki, 1964, and it was approved by the Ethics Committee of Far Eastern Memorial Hospital, New Taipei City, Taiwan (112 143‐F).

### 2.2 Assessment

#### 2.2.1 Assessment of Sarcopenia

Sarcopenia was evaluated by handgrip strength, skeletal muscle mass index (SMI) and physical performance in concurrence with the consensus of the AWGS [16]. Each older participant received a handgrip strength examination on the dominant hand with full elbow extension via a Smedley dynamometer (Takei Ltd., Niigata, Japan). The handgrip strength was measured in kilogrammes (kg). Handgrip strength < 18.0 kg for women and < 28.0 kg for men were classified as low muscle strength [1]. The appendicular skeletal muscle mass (ASM) was estimated by a multifrequency bioelectrical impedance analyser (bc‐418; Tanita, Tokyo, Japan). The acquired ASM was divided by the square of the body height (m^2^) to get the SMI. SMI < 5.7 kg/m^2^ for women and < 7.0 kg/m^2^ for men were defined as low muscle mass [1]. Physical performance was appraised by a 6‐m walk. The participants were requested to walk with a normal pace for 6 m. Gait speed < 1 m/s was categorised as slow gait speed [1]. According to the consensus of AWGS 2019 [1], older adults with low muscle mass and with low muscle strength or low physical performance are diagnosed as sarcopenia; older adults with low muscle mass and both low muscle strength and low physical performance are confirmed as having severe sarcopenia. Low muscle strength older adults with preserved muscle mass were categorised as having “possible sarcopenia.”

#### 2.2.2 Assessment of Kidney Function

The participants with CKD were identified according to the guidelines of Kidney Disease Improving Global Outcomes (KDIGO) [17]. The duration of kidney function or structure abnormalities that persisted for more than 3 months for the diagnosis of CKD was obtained by the participant's self‐report and a medical record review. The blood samples were acquired by veinous punctures. After serum creatinine was measured, creatinine‐based estimated glomerular filtration rate (eGFR) was calculated using the Modification of Diet in Renal Disease (MDRD) Study equation: 186.3 × (serum creatinine value)^‐1.154^ × age^‐0.203^ × (0.742 if female) [18]. The eGFR value was expressed in mL/min/1.73 m^2^. CKD categories were classified based on the guidelines of KDIGO. Participants with eGFR ≥90, 60–89 and < 60 mL/min/1.73 m^2^ were grouped into G1, G2 and G3–5, respectively [17].

#### 2.2.3 Diabetes Status, Comorbidities and Other Variables

The participants' past medical history, including hypertension, ischemic heart disease, DM, depression and osteoporosis, was obtained via face‐to‐face interviews with trained researchers. An additional double‐check was performed through medical records to confirm accuracy. After obtaining blood samples via veinous punctures from DM patients, HbA1c values were measured for evaluating a secondary objective regarding only the diabetes patients. The DM patients with HbA1c < 7% were enrolled in the good control group. The DM patients with HbA1c **≥** 7% were enrolled in the poor control group [19]. Demographic data, such as age, sex and body weight were obtained at the study entry by trained nurses.

### 2.3 Statistical Analyses

Number and percentage were used to present the distribution in categorical variables (including gender, comorbidity and CKD stage) and mean and standard deviation in continuous variables (including age and body weight). The Chi‐square test and ANOVA were used to estimate the difference of categorical and continuous variables, respectively. The Bonferroni test was used for post hoc analysis. Polytomous logistic regression was used to assess the odds ratio (OR) and 95% confidence intervals (CIs) of the sarcopenia stage and sarcopenia‐associated risk factors. Multivariable polytomous logistic regression was adjusted for variables with a significant difference as shown in Table 1. The trend test of sarcopenia stage and sarcopenia‐associated risk factor was examined by multivariable linear regression. The interaction effect of sarcopenia between CKD and diabetes was used in multivariable logistic regression. All statistical tests were two‐sided, and the statistical significance was defined as *p*‐value < 0.05 using SAS, version 9.4 (SAS Institute, Cary, North Carolina).

**TABLE 1:** Distribution of demographics in study subjects.

Variable	Total *N* = 3648		Normal *N* = 2636 (72.3%)		Possible Sarcopenia *N* = 870 (23.9%)		Sarcopenia *N* = 94 (2.58%)		Severe sarcopenia *N* = 48 (1.32%)		*p*‐value
Age, mean (SD)	71.9	(6.07)	70.7	(5.15)	74.7	(7.06)	74.4	(6.49)	80.0	(7.76)	<0.0001^1,2,3,5,6^
Men, *n* (%)	1701	(46.3)	1109	(42.1)	490	(56.3)	68	(72.3)	34	(70.8)	< 0.0001^1,2,3,4^
Comorbidity, *n* (%)											
DM											0.002^1,2,4^
Good control	238	(6.52)	155	(5.88)	74	(8.51)	7	(7.45)	2	(4.17)	
Poor control	615	(16.9)	423	(16.1)	175	(20.1)	10	(10.6)	7	(14.6)	
HTN	1266	(34.7)	893	(33.9)	342	(39.3)	17	(18.1)	14	(29.2)	0.0001^1,2,4^
IHD	140	(3.84)	94	(3.57)	42	(4.83)	4	(4.26)	0	(0.00)	0.187
Depression	88	(2.41)	58	(2.20)	25	(2.87)	1	(1.06)	4	(8.33)	0.027^3^
Osteoporosis	65	(1.78)	39	(1.48)	25	(2.87)	1	(1.06)	0	(0.00)	0.038^1^
CKD stage											< 0.0001^1,3^
G1 (eGFR ≥90)	1391	(38.1)	1041	(39.5)	299	(34.4)	36	(38.3)	15	(31.3)	
G2 (eGFR 60–89)	1776	(48.7)	1303	(49.4)	409	(47.0)	44	(46.8)	20	(41.7)	
G3–5 (eGFR < 60)	481	(13.2)	292	(11.1)	162	(18.6)	14	(14.9)	13	(27.1)	
Weight, mean (SD)	62.6	(11.4)	63.1	(11.3)	62.5	(11.2)	52.7	(8.01)	51.1	(9.43)	< 0.0001^2,3,4,5^

*Note:* 1 missing in body weight (1 in normal). DM with poor control: HbA1c ≥ 7. Post hoc analysis by Bonferroni test. *p* < 0.05 in normal vs. possible Sarcopenia^1^; in normal vs. Sarcopenia^2^; in normal vs. Severe Sarcopenia^3^; in possible Sarcopenia vs. Sarcopenia^4^; in possible Sarcopenia vs. Severe Sarcopenia^5^; in Sarcopenia vs. Severe Sarcopenia^6^.

## 3 Results

**FIGURE 1:**
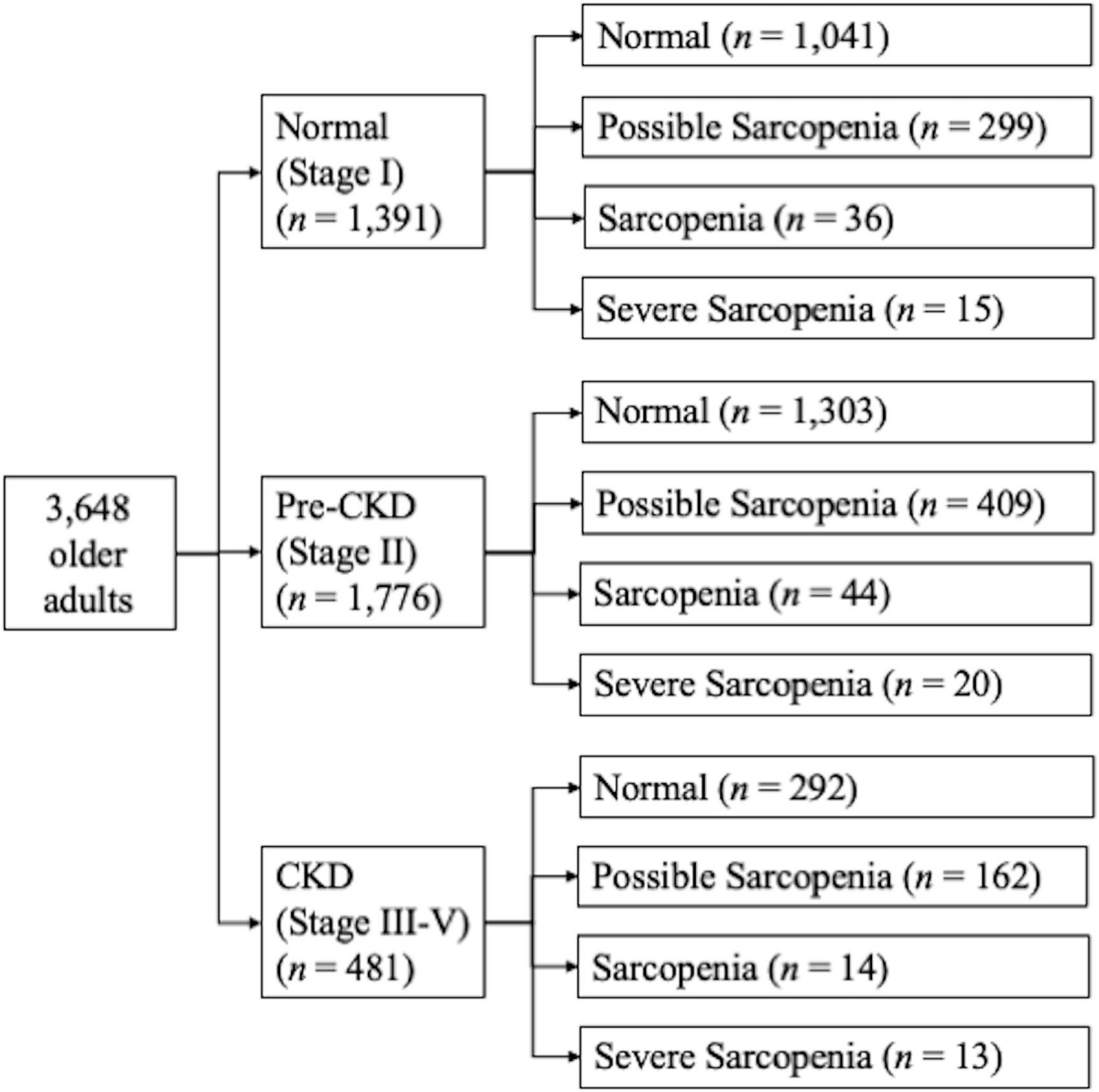
Flow chart of the study participants.

A total of 3648 older subjects were collected in this study, 2636 in normal (72.3%), 870 in possible sarcopenia (23.9%), 94 in sarcopenia (2.58%) and 48 in severe sarcopenia (1.32%) groups. Severe sarcopenia patients were the eldest (80.0 ± 7.76 years old), and normal older adults were the youngest (70.7 ± 5.15 years old) (Table 1). Sarcopenia and severe sarcopenia had a higher proportion of male patients than the normal and possible sarcopenia patients (*p* < 0.0001). Possible sarcopenia patients were more likely to have diabetes (28.6%), hypertension (39.3%), depression (2.87%) and osteoporosis (2.87%) than the normal group. Severe sarcopenia patients were more likely to have CKD (G3–5) (27.1%), but normal subjects were less likely to have CKD (11.1%). Body weight decreased as the sarcopenia stage increased. The detailed subgroup numbers of the study subjects are displayed in Figure 1.

Table 2 shows the risk of the sarcopenia stage and sarcopenia‐associated risk factor by multivariable polytomous logistic regression adjusted for age, gender, diabetes, hypertension, depression, osteoporosis, CKD stage and body weight. Possible sarcopenia, sarcopenia and severe sarcopenia risk increased with ageing (OR = 1.11, 1.10 and 1.23; 95% CI = 1.10–1.13, 1.07–1.15 and 1.18–1.30, respectively). Men had the highest risk of severe sarcopenia (OR = 25.4; 95% CI = 11.3–57.2), followed by sarcopenia (OR = 20.3; 95% CI = 11.5–36.0) and possible sarcopenia (OR = 2.26; 95% CI = 1.87–2.73). The risk factor in possible sarcopenia risk was diabetes, in sarcopenia the risk factor was hypertension and in severe sarcopenia the risk factor was depression. Possible sarcopenia, sarcopenia and severe sarcopenia risk decreased with increasing body weight (OR = 0.98, 0.85 and 0.83; 95% CI = 0.97–0.99, 0.83–0.87 and 0.79–0.86, respectively). The risk for higher sarcopenia status increased with ageing, male gender and depression (*p* < 0.05) (Table 3) but decreased with increasing body weight (*p* < 0.0001).

**TABLE 2:** Adjusted odds ratio of sarcopenia and sarcopenia‐associated risk factor.

	Possible sarcopenia		Sarcopenia		Severe sarcopenia	
Variable	OR (95% CI)	*p*‐value	OR (95% CI)	*p*‐value	OR (95% CI)	*p*‐value
Age	1.11 (1.10–1.13)	< 0.0001	1.10 (1.07–1.15)	< 0.0001	1.23 (1.18–1.30)	< 0.0001
Men vs. women	2.26 (1.87–2.73)	< 0.0001	20.3 (11.5–36.0)	< 0.0001	25.4 (11.3–57.2)	< 0.0001
Comorbidity						
DM						
Good control	1.55 (1.13–2.13)	0.007	1.33 (0.54–3.24)	0.534	0.70 (0.14–3.37)	0.652
Poor control	1.41 (1.13–1.76)	0.002	1.26 (0.61–2.60)	0.539	1.66 (0.66–4.13)	0.280
HTN	1.10 (0.92–1.32)	0.294	0.48 (0.26–0.86)	0.015	0.62 (0.29–1.30)	0.201
Depression	1.34 (0.81–2.23)	0.254	0.80 (0.11–6.14)	0.833	7.64 (2.06–28.4)	0.002
Osteoporosis	1.66 (0.96–2.87)	0.067	0.70 (0.09–5.42)	0.735	NA	
CKD stage						
G2 (eGFR 60–89)	0.84 (0.70–1.01)	0.057	0.78 (0.48–1.26)	0.305	0.59 (0.28–1.25)	0.169
G3–5 (eGFR < 60)	0.97 (0.75–1.26)	0.821	0.88 (0.43–1.78)	0.718	0.91 (0.37–2.27)	0.838
Body weight	0.98 (0.97–0.99)	< 0.0001	0.85 (0.83–0.87)	< 0.0001	0.83 (0.79–0.86)	< 0.0001

*Note:* Manually adjusted for age, gender, diabetes, hypertension, depression, osteoporosis, CKD stage and body weight. The reference groups are women by gender, nondiabetic by DM and G1 by CKD stage.

**TABLE 3:** Association of sarcopenia status and sarcopenia‐associated risk factor in predialysis CKD (G3–5) patients in linear regression.

Variable	Estimated	SE	*t*‐value	*p*‐value
Age, year	0.027	0.002	14.78	< 0.0001
Men vs. women	0.284	0.026	10.63	< 0.0001
DM				
Good control	0.042	0.049	0.86	0.387
Poor control	0.037	0.032	1.15	0.248
HTN	0.015	0.026	0.57	0.571
Depression	0.171	0.073	2.35	0.019
Osteoporosis	0.099	0.090	1.10	0.271
Body weight	−0.013	0.001	−10.82	< 0.0001

*Note:* Manually adjusted for age, gender, diabetes, hypertension, depression, osteoporosis, CKD stage and body weight.

The interaction effect of sarcopenia between diabetes and CKD is presented in Table 4; patients with CKD and diabetes were more likely to develop sarcopenia (34.3%), and patients without CKD and diabetes were less likely to develop sarcopenia (23.5%). Compared to patients without CKD and diabetes, patients with diabetes had a significantly higher sarcopenia relationship in multivariable logistic regression after adjusting for age, gender, hypertension, depression, osteoporosis and body weight (OR = 1.64; 95% CI = 1.21–2.21).

**TABLE 4:** Interaction effect of sarcopenia between DM and predialysis CKD (G3–5).

Variable		Sarcopenia					
CKD	DM	*n*	%	Crude OR (95% CI)	*p*‐value	Adjusted OR (95% CI)	*p*‐value
No	No	254	23.5	Ref.		Ref.	
Yes	No	371	26.4	1.17 (0.97–1.40)	0.101	0.89 (0.73–1.09)	0.247
No	Yes	96	31.1	1.47 (1.11–1.94)	0.007	1.64 (1.21–2.21)	0.001
Yes	Yes	291	34.3	1.70 (1.39–2.08)	< 0.0001	1.11 (0.88–1.40)	0.367

*Note:* Adjusted for age, gender, hypertension, depression, osteoporosis and body weight.

## 4 Discussion

In this study, no correlation was found between predialysis CKD (G3–5) and all three statuses of sarcopenia. The synergistic effect of CKD and DM on the risk of sarcopenia was presented only in the unadjusted model. Compared with the subjects without CKD and DM, DM is a significant independent risk factor for sarcopenia, but there is no remarkable interaction effect between CKD and diabetes on the risk of sarcopenia in older adults after adjustment. It is noteworthy that the male gender and ageing are independent risk factors for all three statuses of sarcopenia, and both factors are attributed to increasing risk of a higher status of sarcopenia in older adults with CKD not yet on dialysis.

The prevalence of sarcopenia varies from 25.6% to 37% in ESKD patients undergoing haemodialysis [5, 20, 21] and from 5.9% to 7.1% in predialysis CKD patients [10, 21, 22]. The MDRD Study and Chronic Kidney Disease Epidemiology Collaboration (CKD‐EPI) formulas, which have shown their clinical accuracy and efficiency in the classification of CKD stages, were applied to evaluate the association between sarcopenia and predialysis CKD in several observations [8, 9, 10, 23, 24, 25, 26, 27, 28, 29]. Regardless of the formula used, most of the studies indicated similar results to our work that the risk of sarcopenia does not increase in predialysis CKD patients [8, 9, 10, 25, 26, 28, 29]. However, the following three studies drew different conclusions from our findings. Kim et al. reported a 3.13‐fold increased risk of sarcopenia only in older male patients with stage III CKD [23]. Moon et al. found a 1.93‐fold increased risk of sarcopenia only in over 40‐year‐old male patients with stages III–V CKD [24]. In a survey by Sharma and colleagues, the relation between sarcopenia and CKD was a U‐shaped distribution with 2.66‐fold and 2.58‐fold increased risk for sarcopenia observed in older adults with eGFR ≥90 and 15–29 mL/min/1.73 m^2^, respectively [27]. Compared to our study, the reasons for this discrepancy may be due to differences in the age of the study subjects, the measurement of muscle quantity and quality, the definition of sarcopenia and ethnicity. However, three recent studies employed the updated diagnostic criteria of sarcopenia similar to AWGS 2019 [10, 26, 29] and mentioned no association between sarcopenia and different CKD stages; our results are in agreement with these studies' conclusions.

Several authors have illustrated the significant association between sarcopenia and DM [4, 30, 31], and CKD is well‐known as a common complication and concomitant condition of DM, but the correlation among sarcopenia, CKD and DM is controversial [11, 26]. Lee and colleagues investigated the synergistic effect of CKD and DM on the risk of sarcopenia in middle‐aged Koreans [11]. Since they defined CKD as eGFR < 60 mL/min/1.73 m^2^ or the presence of albuminuria by dipstick test at baseline examination, the participants with CKD had the higher mean eGFR (71.6 mL/min/1.73 m^2^) and about 96.0% of their participants were CKD I‐IIIa. These authors concluded the synergism of CKD and DM on the risk for sarcopenia. Because temporary albuminuria can be detected in several clinical conditions, such as infection, inflammation and urolithiasis, the albuminuria dipstick test was not administered as a reliable indicator for CKD in our study. In this study, compared to the older adults without DM and CKD, a significant 1.64‐fold increased risk for sarcopenia in the DM of older adults without CKD was noted after adjusting for age, gender, hypertension, depression, osteoporosis and body weight (Table 4). Our results are in agreement with previous studies [11, 26]. Our diabetes older adults with CKD showed a 1.70‐fold increased risk for sarcopenia in the unadjusted analyses, but this synergistic effect between DM and CKD dissipated after multivariable adjustment (Table 4). We suggested that these shared risk factors may contribute to the relatively prominent degree of synergism seen in the unadjusted model. The discrepancy between our work and the report of Lee et al. [11] may be due to the result of differences in the age of the study population and the definition of CKD. Further prospective investigations with optimal CKD diagnostic criteria to evaluate the association among sarcopenia, CKD and DM are needed.

Males were identified to have a higher prevalence of sarcopenia in several previous pieces of research literature [32, 33, 34], but research on the impact of male gender on different statuses of sarcopenia is scarce. In this study, compared with the older female adults, we are not only in agreement with the previous study that the male gender is an independent risk factor of sarcopenia [33, 34] but we also measured the relative association between the male gender and different statuses of sarcopenia and recognised a remarkably increased risk of possible sarcopenia, sarcopenia and severe sarcopenia by 2.26‐fold, 20.3‐fold and 25.4‐fold, respectively, in the older male participants (Table 2). This study firstly reported a significantly higher risk for sarcopenia and severe sarcopenia in older males; we therefore suggested that sarcopenia and severe sarcopenia prevention is relatively important in the older male population. In the subgroup analysis, compared with the older female participants with predialysis CKD, we demonstrated that older males with CKD not yet on dialysis had a 0.284 higher sarcopenia status than the comparisons (Table 3). To the best of our knowledge, this is the first study to elucidate the impact of gender on the risk of sarcopenia statuses in the older male population with predialysis CKD. Prevention and early survey for sarcopenia in older male adults and aggressive management for sarcopenia to prevent the risk of higher sarcopenia status in older male adults with predialysis CKD are recommended.

Although the ageing process is attributed as a crucial part of sarcopenia development, most of the previous studies did not survey the impact of ageing on the different statuses of sarcopenia separately due to a relatively smaller sample size. Our results are in line with these previous findings [9, 23, 33, 34], and we further revealed that ageing could significantly increase risk by 1.11‐fold, 1.10‐fold and 1.23‐fold of possible sarcopenia, sarcopenia and severe sarcopenia, respectively (Table 2). Moreover, this study first exhibited that ageing could significantly increase sarcopenia progression risk by 0.27‐fold every 10 years in patients with predialysis CKD (Table 3). Our results illuminate the importance of the ageing process on the risk of sarcopenia, especially in patients with predialysis CKD. Early identification of sarcopenia and aggressive treatment to prevent disease progression in older adults are essential.

Kidney function may be overestimated by using serum creatinine to calculate eGFR in patients with muscle wasting or reduced muscle mass [35]. This phenomenon may be a potential confounding factor while investigating the association between sarcopenia and predialysis CKD via current commonly used eGFR calculation formulae. Although the CKD‐EPI creatinine equation has been suggested to be more accurate than the MDRD study equation [36], similar to the MDRD study formula, controversial conclusions for the relation between sarcopenia and predialysis CKD by CKD‐EPI equation were reported in previous literature [9, 24, 25, 26, 27, 28, 29]. Two Korean studies have surveyed the association between sarcopenia and predialysis CKD with the MDRD study and CKD‐EPI equations separately [23, 24]. They provided a chance to compare the accuracy between these two creatinine‐based formulae. Since these two studies were conducted with the same database, several potent confounding variables, such as ethnicity, the measurement of muscle quantity and quality and the definition of sarcopenia, could be eliminated. Regardless of the equation applied, both investigations showed similar results that a remarkable correlation between sarcopenia and predialysis CKD was observed only in the subgroup analysis. Altogether, we suggested that the MDRD study and CKD‐EPI equations may provide similar strength while estimating the relationship between sarcopenia and predialysis CKD. Owing to the MDRD study equation being commonly employed in clinical CKD evaluation, and there being fewer publications on the relation between sarcopenia and predialysis CKD estimated by the MDRD study equation, we conducted a large sample‐sized study and revealed no significant association between different statuses of sarcopenia and predialysis CKD estimated by the MDRD study equation.

Other commonly used eGFR formulas, such as Berlin Initiative Study (BIS) and Full Age Spectrum (FAS) equations, presented similar findings with our study results [26, 37]. Formiga et al. stated that the male gender, but not CKD stages, is an independent risk factor for sarcopenia in older patients with predialysis CKD in their study using the BIS equation for CKD categorisation [26]. Only a slight difference was observed in a study comparing the prevalence of sarcopenia in patients with predialysis CKD using several creatinine‐based eGFR formulas, including CKD‐EPI, BIS and FAS, to classify CKD stages [37]. Although our results are consistent with most of the previous literature [8, 9, 10, 25, 26, 28, 29], and the aforementioned huge difference in sarcopenia prevalence between ESKD and predialysis CKD patients also supports our findings [5, 10, 20, 21, 22], decreased serum creatinine levels due to reduced muscle mass in patients with sarcopenia remains a considerable issue; further investigation with a trustworthy renal function measurement method is necessary.

This cross‐sectional study investigated the association between sarcopenia and predialysis CKD. The risk factors for sarcopenia in older patients with CKD not yet on dialysis were also evaluated; it helps to recognise the impact of gender and the ageing process on the risk of sarcopenia development in this population. This work addresses the growing requirement to establish and implement practical approaches to prevent sarcopenia in older adults with predialysis CKD in both prevention and treatment phases. The present report highlights the direction and importance of gender and the ageing process in sarcopenia prevention. We also stated a similar result that no remarkable correlation could be found between sarcopenia and predialysis CDK in older adults, which has been described in previous studies [8, 9, 10, 25, 26, 28, 29].

There are several limitations in this study. Although our findings demonstrated that male gender and age could contribute to the increase of sarcopenia stage progression in older patients with CKD not yet on dialysis, it is difficult to address any causal relationship among the male gender, age, CKD stage and sarcopenia under a cross‐sectional design. Second, the current commonly used creatinine‐based formulae may overestimate the value of eGFR in predialysis CKD patients with sarcopenia [35]. An optimal GFR measurement method to increase the power of detecting the relation between sarcopenia and kidney function is warranted in the future. Third, most of the older participants recruited in this investigation may be in relatively good physical condition, since these volunteers could participate in the community‐based study independently. Older adults with severe comorbidities and lower physical performance may not be enrolled in this survey. For estimating the association between sarcopenia and predialysis CKD, further population‐based studies with less limited inclusion criteria to prevent sample selection bias are suggested. Finally, the accuracy of the bioelectrical impedance analyser for skeletal muscle mass measurement could be impeded by CKD‐related factors, such as fluctuations in the patient's hydration status [38]. Although bioimpedance spectroscopy may have higher accuracy than the bioelectrical impedance analyser, spectroscopy is not recommended to assess skeletal muscle mass in AWGS 2019 [1]. Since BIA is cost‐effective, noninvasive and portable, BIA was applied as the main clinical evaluation method for sarcopenia in the study.

## 5 Conclusions

Although our results presented no interaction effect between DM and CKD on the risk of sarcopenia development, diabetes is a remarkably independent risk factor for sarcopenia in older adults. Moreover, male gender and the ageing process are important risk factors for sarcopenia, and they showed significant association with the increased risk of higher sarcopenia status in older patients with CKD not yet on dialysis. Further prospective large sample size longitudinal cohort studies with optimal CKD staging strategy and less limited inclusion criteria to diminish confounding variables and validate our findings are required in the future.

## Conflicts of Interest

The authors declare no conflicts of interest.
